# Behavioral Control and Reward Sensitivity in Adolescents’ Risk Taking Behavior: A Longitudinal TRAILS Study

**DOI:** 10.3389/fpsyg.2017.00231

**Published:** 2017-02-17

**Authors:** Margot Peeters, Tineke Oldehinkel, Wilma Vollebergh

**Affiliations:** ^1^Interdisciplinary Social Sciences, Utrecht UniversityUtrecht, Netherlands; ^2^University Medical Center Groningen, University of GroningenGroningen, Netherlands

**Keywords:** behavioral control, reward sensitivity, risk taking, substance use, adolescence

## Abstract

Neurodevelopmental theories of risk behavior hypothesize that low behavioral control in combination with high reward sensitivity explains adolescents’ risk behavior. However, empirical studies examining this hypothesis while including actual risk taking behavior in adolescence are lacking. In this study we tested whether the imbalance between behavioral control and reward sensitivity underlies risk taking behavior in adolescence, using a nationally representative longitudinal sample of 715 adolescents, of which 66% revealed an increased risk for mental health problems. To assess behavioral control at age 11 we used both self-report (effortful control) as well as behavioral measures of cognitive control (i.e., working memory and response inhibition). Reward sensitivity was assessed with the Bangor Gambling Task. The main finding of this study was that effortful control at age 11 was the best predictor of risk taking behavior (alcohol and cannabis use) at age 16, particularly among adolescents who were more reward sensitive. Risk taking behavior in adolescents might be explained by relatively weak behavioral control functioning combined with high sensitivity for reward.

## Introduction

The peak in risk taking behavior, assumed to occur in mid adolescence (14–17 years), has received much attention from different fields of research. Recently, neurodevelopmental studies using fMRI techniques have observed developmental disparities in brain regions associated with behavioral control and reward sensitivity, possibly explaining the peak in risk behavior which is typical for mid adolescence (15–17 years, Galvan et al., 2006; Giedd, 2008; Somerville et al., 2011). The results suggest that brain regions associated with reward and cognitive control follow a different developmental trajectory, resulting in fully developed and relatively hypersensitive reward systems (e.g., affective processing) while control systems are still developing until late adolescence (>18–21 years). Although these differences in neurobiological substrates have been found in several studies (Ernst et al., 2005; Galvan et al., 2006, 2007; Silverman et al., 2015) and used to explain the peak in risk taking behavior characteristic of the mid adolescence period (Spear, 2000; Casey et al., 2008), empirical evidence on the assumed interaction effect of behavioral control and reward sensitivity on the actual risk taking behavior of adolescents is scarce. Adolescents who have difficulties in controlling their behavior and are reward sensitive might be more likely to engage in risk behavior, as the motivation to engage in risk behavior might be relatively high while the ability to regulate impulses might be relatively low among these adolescents. In the present longitudinal study, we examined the predictive role of behavioral control and studied the differential effect of reward sensitivity on risk taking behavior in a sample of young adolescents. To the best of our knowledge this is one of the first empirical studies examining the development of risk taking behavior in real life in relation to behavioral control and reward sensitivity.

### Behavioral Control and Risk Behavior

During adolescence, several neurobiological alterations take place, to some extent driven by pubertal changes and hormones (Giedd, 2008; Blakemore et al., 2010; Peters et al., 2015). While some brain areas, such as the visual cortex and motor cortex, are already fully developed in childhood (Gazzaniga et al., 2002), the fine-tuning of other brain regions, such as the prefrontal cortex, is still in progress during adolescence (Luna et al., 2004; Blakemore and Choudhury, 2006). The prefrontal cortex plays a major role in the regulation of behavior. In the present study, we assessed elements of behavioral control including cognitions that are assumed to be involved in the regulation of behavior (Moffitt et al., 2011), such as the inhibition of responses (e.g., response inhibition) and delay of gratification (Krueger et al., 1996), as well as traits and personality characteristics, such as acting without thinking (Barratt, 1983; Evenden, 1999). We assessed two elements of behavioral control; self-reported behavioral control (i.e., effortful control) and cognitive control (i.e., response inhibition and working memory, Peeters et al., 2015).

Several studies from different disciplines (cf. Verdejo-García et al., 2008) have ascertained the role of behavioral control in the onset and continuation of risk behavior. The use of substances among adolescents, for instance, has been linked to problems with delay of responses (Romer et al., 2010), inhibition problems (Fernie et al., 2013), self-reported impulsivity (Krank et al., 2011; White et al., 2011), and effortful control in particular (Piehler et al., 2012). Behavioral control appears to play a vital role in initiating alcohol use (Squeglia et al., 2014; Peeters et al., 2015) and the development of problematic drinking behavior in adolescents (Nigg et al., 2006). Problems with inhibition, both assessed on a cognitive as well as on a behavioral level increase the chance of early initiating of alcohol use and problem drinking in adolescents. Whelan et al. (2012) found reduced activity in brain regions important for cognitive control even among adolescents who just initiated alcohol use, suggesting that vulnerabilities in neural circuits underlying cognitive control might precede the initiation of drinking behavior in adolescents (Wetherill et al., 2013).

Altogether, these results suggest that relatively early weaknesses in behavioral control might place adolescents at risk for early initiation of risk behavior and the development of related problems. Adolescents with behavioral control problems are more likely to act without thinking and less likely delay response, receiving decreased attention for the negative consequences of behavior and increasing their involvement in risk behavior. This reasoning is in line with Krueger et al. (1996) and Tarter et al. (2003) who assumed that early weaknesses in behavioral control underlie the development of externalizing psychopathology later in life.

### Reward Sensitivity and Risk Behavior

Adolescent risk taking behavior is sometimes conceptualized as irrational and impulsive; however, studies suggest little to no differences in risk evaluation and perception between adults and adolescents (Gerrard et al., 1996; Steinberg and Cauffman, 1996; Spear, 2000). This suggests that adolescents, just like adults, are aware of the consequences involved in risk taking behavior. One possible explanation for the observed difference in risk taking behavior among adolescents and adults might thus be that the expected rewarding value of (some) risk behaviors is greater for adolescents than for adults (Van Leijenhorst et al., 2010a,b; Spear, 2011). The gains might simply be much higher for adolescents than for adults when engaging in risky behaviors (Crone and Dahl, 2012). In addition, adolescents might be more sensitive to rewards than adults. Indeed, Chein et al. (2011) found increased activity in reward related brain systems in adolescents while performing a risk taking task; however, this increase was only observed when adolescents completed the task in the presence of peers. This reward sensitization was present not only at a neurocognitive level (e.g., neural activation), but also at a behavioral level (e.g., task performance), resulting in more risk taking behavior by adolescents, as observed by peers. Adults did not reveal this heightened activity or increased risk taking while performing the task with peers (Chein et al., 2011). This study suggested that the peer presence increased the reward for engaging in risky behaviors among adolescents, but not for adults. It seems that the presence of peers changes the perception with respect to the anticipated reward when engaging in risk behavior (Spear, 2011), a change only observed in adolescents but not in adults.

Besides differences in reward perception, the neurological response observed among adults and adolescents is different when faced with the same rewarding stimuli (Ernst et al., 2005; Galvan et al., 2006; Van Leijenhorst et al., 2010a,b). Van Leijenhorst et al. (2010a), for instance, found that adolescents compared to adults and children revealed a heightened neurological response toward rewards in a decision making task. Participants could choose either a low-risk gamble (lower risk and lower reward) or a high-risk gamble (higher risk and higher reward). While performing the task, brain activation was assessed using fMRI techniques. The results indicated that during adolescence, reward related systems show a peak in activation in response to risky decisions, with a possible high rewarding outcome.

With respect to risk taking behavior in real life like substance use, individual variability among adolescents in reward sensitivity either due to heightened neurological responses or higher expected rewarding value of engagement in these risk behaviors might explain why some adolescents more than others engage in risky behaviors like substance use. Taking risk might be more rewarding for some individuals in certain situations compared to different individuals in different situations (Dawe et al., 2004; Galvan et al., 2007). Heightened reward sensitivity might contribute to more risk taking behaviors in reward sensitive adolescents, as shown in a study by van Hemel-Ruiter et al. (2015) who found that adolescents (12–18 years) who scored high on reward sensitivity drank more heavily compared to adolescents who were less sensitive to reward. Moreover, Xiao et al. (2013) found differences in reward sensitivity assessed with the Iowa Gambling Task (IGT; a measure assumed to assess variation in reward sensitivity, Franken and Muris, 2005; Cauffman et al., 2010) between adolescent binge drinking and abstainers, such that binge drinkers were more sensitive for reward than the abstainers. Altogether, these results suggest that individual differences in reward sensitivity are directly associated with risk behaviors, such as alcohol use. Moreover, the increased sensitivity to reward observed in adolescents relative to adults and children affects the level at which behavioral control must be deployed (Somerville et al., 2011).

### Present Study

Neurodevelopmental models suggest that the peak in risk behavior in mid adolescence can be explained by the interplay between not yet fully developed cognitive control functions and increased neural responses toward reward (e.g., dual system models/imbalance model, cf. Spear, 2000; Galvan et al., 2006; Casey et al., 2008; Giedd, 2008; Somerville et al., 2011; Smith et al., 2013; Silverman et al., 2015). To this end it is hypothesized that adolescents with relatively weak behavioral control at age 11 and high reward sensitivity at age 16 are at the greatest risk for risk behaviors at age 16, such as alcohol use, cannabis use, and smoking. To the best of our knowledge, this is the first longitudinal study that examined this interaction looking at both self-reported behavioral control as well as cognitive control. Although these measures all tap in the same underlying construct, namely behavioral control, they might be differently related to specific risky behaviors like substance use (Verdejo-García et al., 2008; Janssen et al., 2015) and interact differently with reward sensitivity.

## Materials and Methods

### Participants

Participants in the present study were selected from a larger longitudinal population study that included 2230 Dutch adolescents who enrolled the study at age 11, and they were followed at least to age 25. For a detailed description of the inclusion criteria and selection process, please see de Winter et al. (2005) and Huisman et al. (2008). Mean age of the population sample was 11.09 years at baseline (*SD* = 0.59, 50.8% girls), 13.56 years at T2 (*SD* = 0.53), and 16.13 years at T3 (*SD* = 0.73). Response rates were 2149 (96.3, 51.2% girls) at T2 and 1816 (81.6, 52.3%) at T3 for the population sample.

At the third wave, a focus sample of 744 adolescents was selected and invited to participate in a number of laboratory tasks. In total, 715 (96.1%) adolescents (49.1% boys) agreed to participate in this experimental session (Mean age T1 = 11.10, *SD* = 0.55, T3 = 16.11, *SD* = 0.59). Adolescents with increased risk for mental health problems (e.g., high frustration/fearfulness and low effortful control, and family risk parental depression, anxiety, addiction, psychoses, antisocial behavior, single parent household) were oversampled in this focus cohort, resulting in a group of 66% adolescents being at risk and 34% adolescents being randomly selected from the population sample (*N* = 715). Information on mental health problems assessed with the Youth Self-Report Scale for the focus sample and for the total TRAILS sample (including simple *t*-test) are provided in **Table 1**. Adolescents in the focus cohort scored significantly higher on Attentional Deficit Hyperactivity Disorder (ADHD) and Oppositional Disorders, in line with Krueger et al. (1996) and Tarter et al. (2003). Due to a strong overlap between these subscales of the YSR and aspects of behavioral control (e.g., ADHD and effortful control Pearson correlation = -0.48, *p* < 0.001; oppositional problems and effortful control Pearson correlation = -0.34, *p* < 0.001) we decided not to include these subscales as confounding variables. The experimental protocol was approved by the Central Committee on Research Involving Human subjects (CCMO). Written informed consent was obtained from the participants. Assessment took place under the guidance of a TRAILS research assistant who received extensive training to ensure a standardized procedure for all participants. Assessment took place at different locations (depending on the residence of the participants). At each location, the experimental room was sound proof, and it had blinded windows (for a detailed description of the procedure of the experimental session, see Bouma et al., 2009).

**Table 1:** Mental health scores and mean differences on subscales of the Youth Self-Report Scale for the total sample and the at-risk sample.

	Mean (SD) risk sample (*N* = 715)	Mean (SD) population Sample (*N* = 2191)	*t*-test
Affective problems	0.300 (0.244)	0.292 (0.247)	-1.595
Anxiety problems	0.354 (0.305)	0.346 (0.305)	-1.131
Somatic problems	0.460 (0.323)	0.458 (0.332)	-0.237
Attentional Deficit Hyperactivity Problems	**0.625 (0.359)**	**0.588 (0.358)**	**-4.822**
Oppositional problems	**0.465 (0.343)**	**0.445 (0.348)**	**-2.660**
Conduct problems	0.241 (0.202)	0.235 (0.197)	-1.465


*Numbers in bold significantly differ from each other.*

In this study, we used a focus cohort, which provided the possibility to include additional measures that might be relevant for this specific group and age. This resulted in varying availability of measures. Measures of behavioral control for instance were available at wave 1, however, not at wave 3, while measures of reward sensitivity were only assessed within the focus cohort and therefore only available at wave 3.

### Measures

#### Risk Behavior

##### Alcohol use

To select the drinking adolescents from the non-drinking adolescents at the first wave, adolescents were asked to indicate whether they ever consumed alcohol in their lives. Adolescents could select from five categories ranging from “never” to “7 times or more.” Depending on their answers to this item, adolescents were selected for the final analyses in which only non-drinkers at wave 1 were included (see also the analyzing strategy). At the third wave, alcohol use was assessed with the quantity by frequency measure (Sobell and Sobell, 1995). Participants indicated on how many days during the week (Monday to Thursday) and weekend (Friday to Sunday, two items) they consumed alcohol on average. In addition, participants were asked to indicate the average number of drinks they consumed on a regular weekend or weekday (two items). The drinking weekdays were multiplied by the number of drinks consumed on a weekday, and the drinking weekend days by the number of drinks on a regular weekend day. A sum score was specified by adding these two numbers together.

##### Cannabis use

At the first wave, adolescents were asked to indicate whether they had ever smoked cannabis in their lives. Adolescents could select from five categories ranging from “never” to “7 times or more.” Depending on their answers to this item, adolescents were selected for the final analyses in which only non-cannabis users at wave 1 were included (see also the analyzing strategy). At the third wave, cannabis use was assessed by the number of occasions (e.g., party, at home, going out) on which cannabis was consumed in the last month. Possible answer categories ranged from 0 to 40 times or more (0–10; 11–19; 20–39; 40 *or more*).

##### Smoking behavior

At the first wave, adolescents were asked to indicate whether they had ever smoked a cigarette in their lives. Adolescents could select from five categories ranging from “never” to “7 times or more.” Depending on their answer to this item, adolescents were selected for the final analyses in which only non-smokers at wave 1 were included (see also the analyzing strategy). At the third wave, adolescents were asked to indicate the amount of cigarettes they smoked per day in the last 4 weeks. Response categories ranged from “never smoked” to “more than 20 cigarettes a day,” with the other categories distinguishing between occasional (e.g., once a week/one per day) and daily smokers (e.g., 2–20 cigarettes per day).

#### Effortful Control

At the first wave (age 11), effortful control was assessed using the child version of the Early Adolescent Temperament Questionnaire revised (EATQ-r; Putnam et al., 2001). This revised version of the EATQ was developed to improve assessment of self-regulation and executive functioning (Putnam et al., 2001). Items loading on the “effortful control” factor were selected to measure self-reported behavioral control. This part of the questionnaire comprises 11 items with response categories ranging from “almost never true” to “almost always true” (e.g., I tend to get in the middle of one thing, then go off and do something else). A Dutch translated version was used (Oldehinkel et al., 2004). The internal consistency of the scale was acceptable (α = 0.69). Higher scores indicate better effortful control.

#### Cognitive Control

At the first wave (age 11), cognitive control was assessed using the Amsterdam Neuropsychology Task (ANT, de Sonneville, 1999). Both working memory and response inhibition, that is, executive functions involved in cognitive control, were assessed with the ANT. In the working memory task, participants have to indicate whether certain letters are presented in the square presented on the computer screen. In the first part (40 trials), the working memory load was low, and participants only needed to indicate whether a certain letter (‘k’) is presented in the square (i.e., yes or no). In the second part (96 trials), the working memory load was high, and the participants needed to indicate whether one of the three letters (‘k, r, s’) are presented on the screen. The median reaction time of the correct trials on the low load (part 1) was subtracted from the median reaction time of the correct trials on the high load (part 3), with higher scores indicating poorer working memory performance. In the response inhibition task, participants received the instruction to indicate on which side the target is located (right or left) by using the corresponding arrows on the keyboard. In the first part (40 trials), the compatible condition, all targets were green, and the participants had to respond congruent with direction of the target. In the second part (40 trials), the incompatible condition, some targets were red, and participants needed to respond in the opposite direction (e.g., left when the target jumps to the right and vice versa). In this latter condition, participants needed to inhibit a predominant response. The median RT in the incompatible condition was subtracted from the compatible condition, with higher scores indicating poorer response inhibition. Both final RT scores were divided by 1000 to avoid large covariances between variables.

#### Reward Sensitivity

In the third wave (age 16), reward sensitivity was assessed using the Bangor Gambling Task (BGT, Bowman and Turnbull, 2004). The BGT is a simplified and alternative version for the IGT (Bechara et al., 1994) assessing responses to reward under arousing circumstances in which real gains and losses can follow behavioral decisions. The BGT uses regular playing cards in which high cards (e.g., jack’s, ace) produce gains in money while low number cards (e.g., 2–10) produce losses. Participants received 5.00 euro (and could keep the money they won), and they were instructed to win as much money as possible by choosing either to “gamble” or “not to gamble” (100 trails). Participants were told that cards were not randomly chosen but specifically selected for this gamble task and that when they would choose wisely they would be able to win money. When participants decided not to gamble, there was no gain or loss of money, regardless of the card. When participants chose the gamble option, they either lost or won the money, depending on the face of that card (win or loss high = /h/h/h0.40, win or loss low = /h/h/h0.20). As the game progressed, the probabilities of losing money increased. To this end it is expected that as the risk to lose money increases with successive blocks, the selection of the non-gamble options should increase accordingly in non-clinical populations. Mean block scores indeed revealed such pattern (mean block is non-gamble – gamble option: mean block 1 = -1.14; mean block 5 = 11.05). Contrary to Bowman and Turnbull (2004), the participants in the present study did not receive more money when they had no money left. This resulted in a situation in which some participants lost all their money after playing 71 cards. To ensure that a gambling score for each participant was calculated in the same manner and based on the same number of cards, only the first 71 played cards were used (the total amount of cards that were played by all participants). The percentage of gambling choices was calculated as the number of gambling choices divided by the total cards played (van Leeuwen et al., 2011), and it was used as a measure of reward sensitivity.

### Strategy of Analysis

First, descriptive statistics and Pearson correlations among the study variables are provided. Second, simple path analyses were used to examine the unique effects of effortful control, cognitive control, and reward sensitivity on the three risk behaviors. For each risk behavior, we selected adolescents who indicated that they had not used the substance in question at baseline. This resulted in three different data sets, one including only non-drinkers at baseline (*N* = 489), one including only non-cannabis users (*N* = 699), and one including only non-smokers at baseline (*N* = 615). **Table 2** includes an overview of the sample size and demographic information for each data set separately. In the second step, interaction variables were created between reward sensitivity and cognitive control and between reward sensitivity and effortful control using centered variables. Each interaction was entered in a separate model in order to maintain a clear interpretation of each interaction effect. Cannabis use and smoking both revealed a skewed distribution with many zeros; therefore, we used a Zero-Inflated Poisson (ZIP) model as a traditional Poisson model is not sufficient when standard deviations are bigger than the mean (over-dispersion; cf. Peeters et al., 2012). The ZIP model allowed us to interpret the continuous part of the model (adolescents who used cannabis/cigarettes) while accounting for the many zero’s. We controlled for gender and used Maximum likelihood with robust standard errors (MLR) as estimation method to account for non-normality of the data in all analysis. FIML was used to deal with missing data. Analyses were completed in Mplus version 7.3 (Muthén and Muthén, 1998). Model fit measures were not informative because all possible paths in the model were estimated (e.g., full model).

**Table 2:** Demographic information and descriptive statistics of study variables for each data set separately.

Data set	N	Mean age wave 1 (*SD*)	% boy	Effortful control T1 Mean (*SD*)	Working memory T1 Mean (*SD*)	Response inhibition T1 Mean (*SD*)	Reward sensitivity T3 Mean (*SD*)
Total sample	715	11.10 (*0.55*)	49.1	3.54 (*0.52*)	0.47 (*0.25*)	0.19 (*0.15*)	0.50 (*0.13)*
Non-alcohol use	486	11.06 (*0.55*)	43.3	3.59 (*0.53*)	0.46 (*0.24*)	0.19 (*0.15*)	0.51 (*0.13*)
Non-cannabis use	699	11.10 (*0.55*)	48.4	3.55 (*0.52*)	0.47 (*0.25*)	0.19 (*0.15*)	0.51 (*0.13*)
Non-smoking behavior	615	11.09 (*0.55*)	47.8	3.59 (*0.52*)	0.46 (*0.26*)	0.20 (*0.16*)	0.50 (*0.14*)


## Results

### Information on Subsamples

For each risk behavior (i.e., alcohol, cannabis, smoking), we selected the non-users at baseline resulting in three different samples with non-users at baseline (either non-drinker, non-cannabis user or non-smoker at wave 1). Measures of behavioral control at wave 1 therefore preceded risk behavior. **Table 2** includes information on mean age, percentage boy/girl, the three measures of behavioral control and for reward sensitivity for the three subsamples separately and for the total sample. We further looked at the use of other substances in the specific subsamples (alcohol, cannabis, and smoking behavior). In the alcohol sample only one adolescent reported cannabis use. Ninety-three percent reported that they never smoked a cigarette (1.5% adolescents indicated cigarette use more than once). In the cannabis subsample, 69.6% reported no alcohol use at baseline (around 15% of the drinkers reported that they only drank alcohol once in their lives). 87.9% reported no smoking behavior (4.5% of the smokers reported cigarette use more than once). In the smoking subsample, 73.8% reported no alcohol use at baseline (around 12% of the drinkers reported that they only drank once in their lives). Only three (0.5%) adolescents reported cannabis use, of whom 1 reported cannabis use more than once at baseline.

### Descriptive Statistics

In **Table 3**, descriptive statistics for the three risk behaviors, alcohol, cannabis, and smoking, are presented. All three risk behaviors revealed a positive association with each other. Furthermore, Pearson correlation revealed poorer effortful control, working memory performance, and response inhibition for boys. Alcohol and cannabis use were both higher among boys; however, smoking behavior appeared to be higher among girls. *T*-test supported this assumption [*t*(684) = 2.847, *p* < 0.005]. In addition, weaker effortful control at T1 was associated with more alcohol use, cannabis use, and smoking behavior at T3. Reward sensitivity at T3 was positively associated with alcohol use at T3, however, no significant correlation was found with cannabis use or smoking. Working memory and response inhibition correlated positively with each other. A negative correlation was found between working memory and effortful control, suggesting poorer working memory functioning is associated with relatively weaker effortful control skills (note that higher scores on inhibition and working memory indicate poorer functioning).

**Table 3:** Descriptive statistics and Pearson correlations for all study variables for the total sample.

	M *(SD)*	1	2	3	4	5	6	7
(1) Boy (%)	49.1%							
(2) Effortful control T1	3.54 *(0.52)*	-0.11^∗^						
(3) Working memory T1	0.47 *(0.25)*	0.16^∗∗^	-0.09^∗^					
(4) Response inhibition T1	0.19 *(0.15)*	0.04	0.01	0.14^∗∗^				
(5) Reward sensitivity T3	0.51 *(0.13)*	-0.01	-0.11^∗^	-0.01	0.04			
(6) QF T3	5.92 *(7.15)*	0.13^∗^	-0.15^∗∗^	0.01	-0.02	0.14^∗∗^		
(7) Cannabis use T3	1.40 (*5.73*)	0.15^∗∗^	-0.15^∗∗^	0.08^∗^	-0.04	0.01	0.28^∗∗^	
(8) Smoking T3	1.56 *(2.37)*	-0.12^∗∗^	-0.12^∗∗^	-0.01	-0.04	0.08	0.48^∗∗^	0.31^∗∗^


*QF, quantity by frequency alcohol use; ^∗^ = *p* < 0.05, ^∗∗^ = *p* < 0.01. Effortful control, higher scores indicate better control; working memory + inhibition, higher scores indicate worse performance/lower control.*

### Main Effects

In **Tables 4**–**6**, the main unique effects of effortful control, cognitive control, and reward sensitivity on risk behavior are presented. We included all three behavioral control measures in the same model to analyze their unique contribution to risk behavior. With respect to alcohol, effortful control at T1 significantly predicted T3 alcohol use (β = -0.15, *SE* = 0.05). That is, adolescents with relatively poor effortful control at T1 increased stronger in their alcohol use between T1 and T3 compared to adolescents with relatively good effortful control. For reward sensitivity and cognitive control, no main effect on alcohol use was found.

**Table 4:** Regression coefficients for main effects, with alcohol use at T3 as outcome measure.

	*B*	*SE*	*p*-value (2-tailed)
**Multivariate main effects**			
Sex	0.09	0.05	0.06
Working memory T1	-0.09	0.05	0.87
Response inhibition T1	-0.01	0.04	0.76
Effortful control T1	-0.14	0.05	<**0.01**
Reward sensitivity T3	0.12	0.08	0.13
**Interaction effects**			
Work × reward	0.33	0.29	0.26
Inhibition × Reward	0.01	0.45	0.97
Effort × Reward	-0.57	0.15	**<0.01**


*Work, working memory; Inhibition, response inhibition; Effort, effortful control; Reward, reward sensitivity. Numbers in bold indicate significant effects.*

**Table 5:** Regression coefficients for main effects, with cannabis use (zero inflated) at T3 as outcome measure.

	*B*	*SE*	*p*-value (2-tailed)
**Multivariate main effects**			
Sex	0.43	0.18	**0.02**
Working memory T1	0.43	0.15	**<0.01**
Response inhibition T1	-0.44	0.19	**0.02**
Effortful control T1	-0.57	0.12	**<0.01**
Reward sensitivity T3	-0.22	0.18	0.24
**Interaction effects**			
Work × reward	-0.64	0.45	0.16
Inhibition × Reward	-0.11	1.16	0.92
Effort × Reward	-1.31	0.46	**<0.01**


*Work, working memory; Inhibition, response inhibition; Effort, effortful control; Reward, reward sensitivity. Numbers in bold indicate significant effects.*

**Table 6:** Regression coefficients for main effects, with smoking (zero inflated) at T3 as outcome measure.

	*B*	*SE*	*p*-value (2-tailed)
**Multivariate main effects**			
Sex	0.07	0.47	0.88
Working memory T1	-0.61	0.36	0.09
Response inhibition T1	-0.34	0.50	0.50
Effortful control T1	-0.44	0.45	0.34
Reward sensitivity T3	0.53	0.44	0.22
**Interaction effects**			
Work × reward	1.26	1.25	0.31
Inhibition × Reward	2.67	1.28	**0.04**
Effort × Reward	-1.84	2.99	0.54


*Work, working memory; Inhibition, response inhibition; Effort, effortful control; Reward, reward sensitivity. Numbers in bold indicate significant effects.*

With respect to cannabis use, a similar pattern for effortful control was observed in that effortful control at T1 predicted a stronger increase in cannabis use between 11 and 16 years of age (β = -0.57, *SE* = 0.12). Weaker effortful control skills at age 11 predicted cannabis use at age 16. In addition, working memory functioning at age 11 predicted cannabis use at age 16 (β = 0.43, *SE* = 0.15). Adolescents with relatively weaker working memory skills at age 11 used more cannabis use at age 16 (note higher scores on working memory indicate poorer functioning). In contrast to what was expected, response inhibition was a significant predictor of cannabis use, with those having relatively good inhibitions skills progressing more heavily in the use of cannabis compared to those with weaker inhibition skills (β = -0.08, *SE* = 0.03). Additional analysis revealed that response inhibition was only a significant predictor of cannabis use when controlling for other measures of behavioral control and not when analyzed alone (β = -0.36, *SE* = 0.40). In addition, gender was a significant predictor of cannabis use at age 16, with boys more likely using cannabis compared to girls.

With respect to smoking behavior, none of the hypothesized main effects were significant. Only gender was a significant unique predictor.

### Differential Effects

**FIGURE 1:**
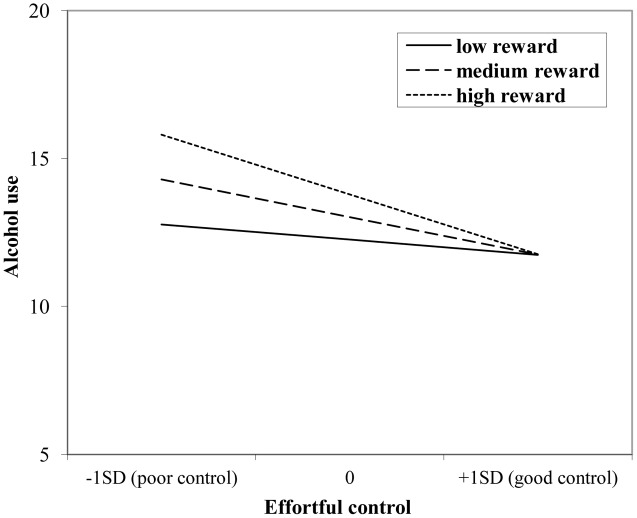
**Interaction of reward sensitivity on the relation between effortful control and alcohol use**.

**FIGURE 2:**
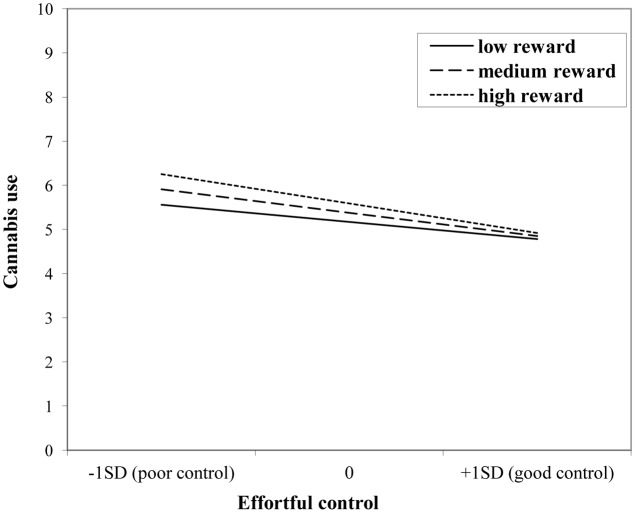
**Interaction of reward sensitivity on the relation between effortful control and cannabis use**.

**FIGURE 3:**
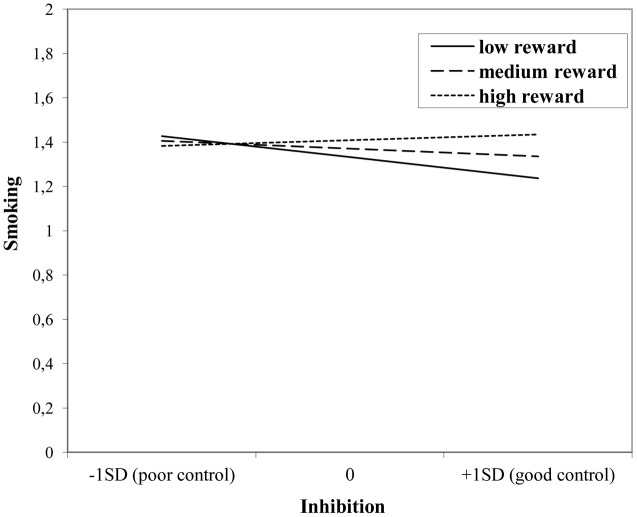
**Interaction of reward sensitivity on the relation between response inhibition and smoking**.

The interaction effect with reward sensitivity was examined for all three measures of behavioral control (**Tables 4**–**6**). A significant interaction was found between reward sensitivity and effortful control for alcohol use (β = -0.57, *SE* = 0.15) and cannabis use (β = -0.28, *SE* = 0.13). These interaction effects are illustrated in **Figures 1** and **2**. The interaction effect reveals that adolescents with relatively poor effortful control at age 11 and high levels of reward sensitivity at age 16 are the heaviest drinkers and cannabis users at age 16 (controlled for previous use). For adolescents with good effortful control at baseline, the level of reward sensitivity in mid adolescence does not appear to influence the amount of alcohol or cannabis that is consumed in mid adolescence. In contrast, for smoking no main effect of response inhibition on smoking behavior was observed; however, the interaction between response inhibition and reward sensitivity predicting smoking behavior was significant (**Figure 3**; β = 2.67, *SE* = 1.28). When response inhibition was relatively good, adolescents who were less reward sensitive smoked less at age 16 (reversed effect).

## Discussion

This study tested the unique and differential effects of behavioral control and reward sensitivity on risk taking behavior among adolescents. The results indicated that effortful control in early adolescence (age 11) was a significant unique predictor of alcohol and cannabis use in mid adolescence (>4 years later). Adolescents with weak effortful control present before alcohol or cannabis use is initiated, progress more strongly in their use of alcohol and cannabis compared to adolescents with relatively good behavioral control. This effect was strongest among adolescents who were relatively more reward sensitive at age 16. It should be noted, however, that the relation between reward sensitivity and substance use is cross-sectional of nature; both outcomes were assessed at age 16. It is possible that substance use at earlier ages results in more reward sensitivity at age 16, and not otherwise. Though, recent findings of Peeters et al. (2013) suggest that motivational processes such as reward sensitivity, more likely predict increase in substance use than that they increase as a result of substance use. Moreover, the findings are in line with Kim-Spoon et al. (2016) who found that reward sensitivity, was associated with earlier substance use onset in the group of adolescents with lower behavioral control. Nevertheless, more longitudinal studies in different cultures (see for an overview Duell et al., 2016) are needed before any firm conclusion can be drawn with respect to the nature of this relationship.

Cognitive control as measured with neurocognitive tasks, predicted only cannabis use at age 16. Adolescents with relatively weak working memory functioning at age 11 were more likely to increase in their cannabis use between 11 and 16 years of age. In addition, and in contrast to the other findings, adolescents with better inhibition skills at age 11 were more likely to increase stronger in cannabis use between 11 and 16 years of age. This surprising finding might be explained by the fact that all measures of behavioral control show overlap, as this relation was only significant when controlling for effortful control and working memory, not when examined alone. Working memory remained a significant predictor of cannabis when analyzed in the absence of other behavioral control measures. Nevertheless, the correlations between the measures of behavioral control were not all significant suggesting that other explanations are needed.

These findings are partly in line with research, suggesting that risk behavior is a result of different neurodevelopmental trajectories, underlying processes of reward, and behavioral control (Galvan et al., 2006; Blakemore and Robbins, 2012). Several researchers have suggested that (emotional) decision making develops in mid adolescence; however, not fully developed control systems could exert insufficient influence on affective processes, resulting in hypersensitivity to reward and increased engagement in risk behavior during adolescence (Steinberg, 2007; Casey and Jones, 2010; Spear, 2011; Crone and Dahl, 2012). Surprisingly, we only found this interaction for self-reported effortful control and not for cognitive control as measured with neurocognitive tasks while according to several theoretical studies (Steinberg, 2007; Casey et al., 2008; Spear, 2011) particularly cognitive control systems would reveal immature development and possibly interact with processes involved in reward. The current study revealed only an effect of cognitive control on cannabis use and not on alcohol use or smoking behavior. It should be noted, however, that cognitive control was assessed in early adolescence (11–12 years) and not in mid adolescence (15–16 years), which is assumed to be the period at which risk taking behavior reaches its’ peak. It is possible that immature brain development, as indicated by these measures of cognitive control, does not necessarily explain the increased risk behavior among adolescents. Somerville et al. (2011), for instance, found different cognitive control responses toward appetitive stimuli among adolescents compared to adult and children. These appetitive stimuli can be seen as rewarding. The results of Somerville et al. (2011) suggested a kind of context-dependent reduced control observed in adolescents who are faced with rewarding stimuli, which has not been seen in adults and children. Similarly, Botdorf et al. (2016) found that weaker behavioral control under arousing circumstances was associated with (laboratory) risk taking behavior. This might indicate that the situation itself elicits impaired cognitive control responses and that it is not so much a matter of immature cognitive control, but rather temporary weaker control in response to rewarding stimuli. Traditional measures of cognitive control (e.g., inhibition/interference tasks, working memory load tasks) might not be able to assess these temporary impairments, as these measures assess cognitive control in so called “cold situations” (Metcalfe and Mischel, 1999). The effortful control scale (e.g., EATQ-R) includes references to so called “hot situations” (e.g., the more I try to stop myself from doing something I shouldn’t, the more likely I am to do it”) and for that reason, it might better tap into behavioral control processes under arousing circumstances. Similarly, Romer et al. (2010) suggested that a rise in sensation seeking might explain the increased risk behavior in mid adolescence. Romer found no difference in the importance of behavioral control in explaining risk behavior, despite the age differences in the sample (e.g., 14–22 years). This implies that irrespective of age, behavioral control is an important predictor of risk behavior and according to Romer et al. (2010), the maturation of cognitive control in adolescence might be less important in explaining risk behavior than previously assumed. In addition, Romer et al. (2010) suggested that experience with risk taking behaviors might even increase behavioral control, as the negative consequences of these behaviors may act as a constrain. In other words, experience with risk behaviors might eventually result in increased control over behavior according to negative reinforcement principles. In the present study, we only looked at behavioral control at the age of 11, before the critical period of 16 years during which a peak in risk behavior is observed. To test this hypothesis in more detail, future research should examine possible increases in behavioral control after involvement in risk behavior in a research design with multiple measurement waves over a closer period of time.

In contrast to what was expected, we did not find a main effect of reward or behavioral control on smoking behavior at age 16. An interaction was found, although in a different direction: Adolescents with good inhibition skills who were low in reward sensitivity indicated less cigarette use. A possible explanation may be that adolescents experience craving for and withdrawal symptoms of smoking differently compared to other risk behaviors, such as alcohol use (Chung and Martin, 2005). Accordingly, behavioral control and reward sensitivity might predict experimental use of smoking but are less successful in predicting regular smoking behavior.

### Limitations

Bedsides the strengths of the study, such as a large sample size and the use of different measures of control, some limitations should be mentioned. First, both effortful control and cognitive control were assessed only at wave 1 at age 11. This allowed us to look at weaknesses in behavioral control before initiation of alcohol. Yet, it can be argued that levels of behavior control at this stage of live are not indicative of levels of control during mid adolescence when the peak in risk taking is observed. After all, the ongoing maturation of the prefrontal cortex during adolescence is assumed to play a vital role in explaining the risk behavior (Steinberg, 2007; Casey and Jones, 2010). At the same time, recent studies have suggested (Forbes and Dahl, 2010; Peters et al., 2015) that the onset of puberty entails hormonal changes that underlie structural brain maturation and influence cognitive processing associated with reward, motivation, and risk taking behavior. Moreover, individual differences in cognitive control might already be visible at this early age, reflecting a general pattern of growth that is not age specific (Spear, 2000; Casey et al., 2008). In addition, longitudinal assessment of cognitive control in the TRAILS study does reveal correlation between cognitive control at ages 11 and 19 (Boelema et al., 2015). Nevertheless, for future research, it would be interesting to include measures of cognitive control in mid adolescence when the peak in risk taking behavior is observed.

Second, the measures of cognitive control in the present study were designed to reveal neurocognitive abnormalities in complex cognitive functioning (de Sonneville, 1999). This measure might be particularly relevant for clinical populations, but it might be less sensitive when it involves detecting differences in functioning in a relatively normal sample of adolescents (Boelema et al., 2015). This might explain why no predictive effects of cognitive control on alcohol were found. Other tasks, such as the Self Ordered Pointing Task might be better in detecting working memory difficulties in non-clinical populations (see for instance Peeters et al., 2015). Third, the BGT has been originally developed to assess decision-making behavior under arousing circumstances (reward-based decision-making). It is possible that the BGT task does not assess reward sensitivity but rather decision making in arousing situations. Nevertheless, the IGT, a similar decision-making task as the BGT, assesses the extent to which immediate rewards are weighted in relation to long term consequences (Ernst et al., 2003; Bechara, 2005), which can be interpreted as a measure of sensitivity to reward (e.g., for some, an immediate reward might not outweigh long term consequences while for others, immediate reward is much more appealing). Similar gambling tasks have been used to examine reward processing at a neuropsychological level (Van Leijenhorst et al., 2010a). In addition, relatively poor performance on the IGT (predominantly preference for immediate gains) has been associated with self-reported reward sensitivity (Davis et al., 2007). A limitation related to the BGT is that only the first 71 cards were used instead of all 100 cards as in Bowman and Turnbull (2004). Nevertheless, the gambling ratio in the first and last blocks in our study revealed similar results (more gambling in the first block, and less gambling options in the last block) as the task used in Bowman and Turnbull (2004). Lastly, since temperament and parental psychopathology were selection criteria for this subsample (cf. Bouma et al., 2009), generalizability of results to other adolescent populations might be restricted. It should be noted that 34% of this sample was selected from the normal population, resulting in a sample slightly oversampled with adolescents at risk for behavioral and mental health problems. Simple *t*-test revealed only significant differences for ADHD and Oppositional Disorder assessed with the Youth Self-Report Scale (see **Table 1** for more details).

## Conclusion

The present study reveals that behavioral control is an important predictor in adolescent risk taking behavior. Adolescents who are reward sensitive and have difficulties in controlling their behavior appear to be most susceptible involvement in risk behavior. The increased susceptibility for reward might encourage some adolescents to explore opportunities and take on challenges, which might be important for the social and emotional development (Forbes and Dahl, 2010; Crone and Dahl, 2012). However, this motivational orientation toward reward might require more control over impulses than present among adolescents who experience problems with behavioral control. As a result, some adolescents might encounter difficulties in regulating their behavior when it involves risk taking behavior while for others, these difficulties might have severe consequences on their (later) health behavior.

## Author Contributions

MP was responsible for the design, analyses, coordination, and draft of the manuscript; WV and TO participated in the design and interpretation of the data and results. All authors read, revised, and approved the final manuscript.

## Conflict of Interest Statement

The authors declare that the research was conducted in the absence of any commercial or financial relationships that could be construed as a potential conflict of interest.
